# Retraction Note: Polyphenols from Conyza dioscoridis (L.) ameliorate Alzheimer’s disease- like alterations through multi-targeting activities in two animal models

**DOI:** 10.1186/s12906-023-04111-8

**Published:** 2023-08-03

**Authors:** Adel A. Gomaa, Hanan S. M. Farghaly, Rania M. Makboul, Abeer M. Hussien, Mariam A. Nicola

**Affiliations:** 1 Department of Pharmacology, Faculty of Medicine, Assiut University, Assiut, Egypt; 2 Department of Pathology, Faculty of Medicine, Assiut University, Assiut, Egypt; 3 Department of Pharmacology and Toxicology, Faculty of Pharmacy, Assiut University, Assiut, Egypt

**BMC Complementary Medicine and Therapies (2022) 22:288**

10.1186/s12906-022-03765-0

The Editor has retracted this article. The T2D + CD100 and T2d + CD150 panels in Fig. 2a appear to partially overlap. The authors have provided the original images upon request; however, these images also appear to overlap.

Additionally, the NC + veh, T2D + veh and T2D + DON images and data in Fig. 2 appear highly similar to the same groups in Fig. 2 of the authors’ earlier article [1].

The Editor therefore no longer has confidence in the reliability of the data and conclusions reported in this article.

Adel A. Gomaa, Hanan S. M. Farghaly and Mariam A. Nicola do not agree to this retraction. Rania M. Makboul and Abeer M. Hussien have not responded to any correspondence from the Editor about this retraction.
